# Dark patterns in online gambling: A scoping review and classification of deceptive design practices

**DOI:** 10.1556/2006.2025.00096

**Published:** 2026-01-06

**Authors:** Jack McGarrigle, Jessica Smith, Joe Griffiths, Jamie Torrance, Martyn Quigley, Simon Dymond

**Affiliations:** 1 School of Psychology, Swansea University, United Kingdom; 2 Department of Psychology, Reykjavik University, Iceland

**Keywords:** dark patterns, dark nudges, sludge, gambling, platform design, consumer protection

## Abstract

**Background and aims:**

Dark patterns are online platform design features that influence consumer behaviour to the advantage of the interface designer. In online gambling, such designs may exacerbate gambling-related harms, particularly among vulnerable consumers. This study aims to provide the first scoping review of dark patterns in online gambling.

**Methods:**

Following established scoping review frameworks, we systematically searched databases and grey literature using terms related to dark patterns and online gambling. The review protocol was preregistered.

**Results:**

Included articles (*n* = 16) addressed a variety of gambling-related dark patterns: hidden gambling management tools, inducements with complex conditions, minimum balances required to withdraw funds, unnecessary frictions involved in closing an account, high defaults in stake, deposit, reality check and deposit limit settings, and urgency-based gambling prompts. To address inconsistent terminology across studies, we synthesised existing literature by mapping identified dark patterns to a transdisciplinary framework, providing greater conceptual clarity and direction for future research.

**Discussions and conclusions:**

The potential for harm from dark patterns is evident, yet evidence on behavioural impacts is limited, hindered by restricted access to proprietary gambling operator data. Research in this area is sparse and fragmented, often using inconsistent terminology. Future studies should empirically investigate the influence of dark patterns on consumer behaviour, especially among vulnerable populations, and evaluate safer design alternatives. We recommend mandating gambling operators to collaborate with researchers to assess platform safety, and shifting the burden of proof onto operators to demonstrate that their platforms prioritise consumer safety and foster responsible gambling environments.

## Introduction

Online gambling is a rapidly expanding industry, driven by increased accessibility and regulatory liberalisation. Global online gambling consumer losses are estimated at USD$75 billion annually (Polaris Market Research, 2022), while 71 million adults worldwide are estimated to have experienced harm from gambling in the past year (Tran et al., 2024). As online gambling platforms continue to grow and evolve, understanding the influence of platform designs on consumer behaviour is crucial in informing regulation and ensuring consumer protection.

There is widespread acceptance of a public health approach to gambling harm prevention, in which population-level interventions such as the regulation of systems, products, and environment are prioritised over the traditional focus upon individual behaviour (Wardle et al., 2024). While individual behaviour remains an important component of gambling-related harm, the gambling environment itself warrants equal attention in both policy interventions and academic research, as it may contribute to or exacerbate the behaviours which lead to gambling harm (Reith & Wardle, 2022). In regulating the ever-evolving online gambling landscape, policymakers require a clear understanding of gambling platform designs and their influence on user behaviour.

Technological advancements in the online gambling user experience have increased the potential for consumer harm in comparison with traditional analogue gambling (Marionneau, Ruohio, & Karlsson, 2023). Mobile gambling applications (apps) enable instant access to unlimited live sports betting and casino gambling opportunities at any time of the day (Hing et al., 2024), while consumers are profiled through predictive algorithms and targeted with personalised marketing strategies (Guillou-Landreat, Gallopel-Morvan, Lever, Le Goff, & Le Reste, 2021; Singer, Wöhr, & Otterbach, 2024). The nature of sports betting in particular has been transformed from a discontinuous to a continuous activity, due to technological advancements, instant accessibility, and several key design features. Instant cash out, which allows bettors to settle their stake on an event before it has concluded, can prolong gambling sessions and creates an illusion of control (Lopez-Gonzalez & Griffiths, 2017). Instant depositing of funds facilitates loss-chasing, while the ability to bet on micro-events in-play (e.g. corners, yellow cards) increases the number of betting opportunities and the frequency of outcomes, creating a more rapid and continuous gambling environment, further amplifying the risk of gambling harms (Parke & Parke, 2019; Torrance, O’Hanrahan, Carroll, & Newall, 2024).

The discipline of behavioural economics provides a practical framework with which to analyse the design, choice architecture, and user experience of online gambling platforms (Newall, 2019). Building upon insights from cognitive psychology, the behavioural economics literature has demonstrated that humans decision making often diverges from rationality, and that choices are heavily influenced by the manner in which they are presented (Simon, 1955; Tversky & Kahneman, 1974). These insights form the basis of nudge theory, which involves making subtle changes to choice architecture design, with the goal of influencing decision making in a consistent and predictable way, ultimately improving the overall welfare of the decision maker (Sunstein & Thaler, 2009). Nudges have been widely adopted in public policy, with dedicated “nudge units” in government producing demonstrable improved outcomes in areas such as organ donation, tax compliance, and pension contributions (Hallsworth, List, Metcalfe, & Vlaev, 2017; Halpern & Sanders, 2016). However, in recent years an off-shoot of nudge theory known as “dark nudges” has emerged, in which the same behavioural principles are instead used to exploit users' cognitive biases, steering them toward choices that do not serve their best interests (Newall, 2019; Petticrew, Maani, Pettigrew, Rutter, & Van Schalkwyk, 2020). Closely related is the concept of “sludge”, which refers to excessive frictions experience by consumers which restrict their ability to perform their intended actions (Sunstein, 2022).

In the interests of reducing definitional complexity, within the present review dark nudges and sludge can be understood as existing within an overarching framework, known as dark patterns. Dark patterns are user interface designs which influence behaviour away from the intentions and best interests of users and towards that preferred by the designer of the interface (Cara, 2019; Gray, Sanchez Chamorro, Obi, & Duane, 2023). A well-documented example can be found in online data privacy consent banners where the “accept all” option is made visually prominent using bright colours while the “reject all” option is downplayed in size and colour (Bösch, Erb, Kargl, Kopp, & Pfattheicher, 2016; Nouwens, Liccardi, Veale, Karger, & Kagal, 2020; Soe, Nordberg, Guribye, & Slavkovik, 2020). Other dark pattern design features include pre-selected defaults of consent, as well as the obfuscation or removal of ‘reject’ buttons entirely. Such designs have been found to have a substantial influence on user acceptance of personal data disclosure (Utz, Degeling, Fahl, Schaub, & Holz, 2019).

Dark patterns have been documented in online shopping (Mathur et al., 2019), social media usage (Schaffner, Lingareddy, & Chetty, 2022), mobile apps (Di Geronimo, Braz, Fregnan, Palomba, & Bacchelli, 2020), and video games (Zagal, Björk, & Lewis, 2013). By so doing, they have attracted the attention of international regulatory bodies. Indeed, the term “dark patterns” was codified into EU (European Union, 2022) and US (California Department of Justice, 2022) law, and several public bodies have published guidance (Competition and Markets Authority, 2022; European Commission et al., 2022; Federal Trade Commission, 2022a), and sought fines (Federal Trade Commission, 2022b; Gambling Commission, 2023) for companies who have used dark patterns to deceive or manipulate their consumers.

At present, there are few guidelines or restrictions on product design practices in online gambling. As technological sophistication of the gambling industry continues to grow, there is legitimate concern regarding the ability of regulators to keep pace with new developments (Wardle et al., 2024). As more new users move increasingly towards online gambling platforms (Gambling Commission, 2024a) and as these platforms become more immersive and sophisticated in their behaviourally informed designs, there is a pressing need for an up-to-date understanding of how specific design features influence consumer behaviour. Moreover, it is critical to examine how the influence of dark patterns may vary across key demographic groups, in order to protect vulnerable consumers from gambling harm.

Recently, Newall (2025) highlighted deceptive design features on online gambling platforms as an important yet under-researched topic. Drawing on concepts from behavioural science, they outline a taxonomy distinguishing between “sludge,” “dark patterns,” and “dark nudges,” and call for greater scholarly and regulatory attention to these design practices. Notably, Newall's commentary observed that much relevant evidence may be found in grey literature and therefore left uncovered by systematic database searches. The present study addresses this gap by presenting the first systematic and preregistered scoping review of dark patterns in online gambling research, incorporating both peer-reviewed and grey literature. Our aim is to provide a comprehensive overview of the current state of knowledge in this area by: (1) analysing the characteristics of the studies conducted; (2) identifying the types of dark patterns documented to date on online gambling platforms; (3) examining the terminology used to describe these practices; and (4) summarising findings on their potential behavioural impacts. By highlighting knowledge gaps, this review should complement and extend existing commentary on gambling-related dark patterns, and can help to guide future research direction and regulatory priorities.

## Methodology

This scoping review follows the methodological frameworks laid out by Arksey and O’Malley (2005) and Levac, Colquhoun, and O’Brien (2010). A scoping review has a broader scope than that of a literature or systematic review, and includes grey literature in identifying and synthesising a comprehensive evidence base on topics with limited empirical research to date (Munn et al., 2018). Grey literature is particularly common in gambling research, and as such it is generally recommended to include such research in scoping reviews (Baxter, 2022; Baxter, Nicoll, & Akçayir, 2021). The review protocol was preregistered via the Open Science Framework (OSF) before the search strategy commenced (preregistration URL: https://osf.io/z7vr5/?view_only=36665079362c4d46b550bf76fb6e8beb).

### Data sources and search strategy

A literature search was performed of Scopus, PsychInfo, and PubMed databases. In addition, the websites of 29 grey literature institutions relevant to gambling research were searched for material. These institutions were gathered from a search strategy identified in a joint report by Citizens Advice and the Behavioural Insights Team (2024) (Appendix Table A1). The following search terms were used in conjunction with Boolean operators (AND/OR); *‘dark patterns’, ‘dark nudges’, ‘sludge’, ‘choice architecture’, ‘persuasive design’, ‘gambling’*. These terms were selected to account for the terminological diversity in the literature describing deceptive interface design. ‘Dark patterns’ and ‘Dark nudges’ are often used interchangeably in deceptive design literature and align closely with the focus of this review. ‘Sludge’ was included due to its growing use in behavioural public policy literature to describe frictions impeding consumer choice. ‘Choice architecture’ and ‘persuasive design’ represent broader conceptual categories and were included to capture relevant records which describe dark pattern-like practices without using the exact terminology. Inclusion of these broader terms was deemed necessary given the lack of standardised terminology in the field. The reference lists of included studies were manually checked for additional records which may have fulfilled inclusion criteria.

### Study selection and data extraction

**Fig. 1.**
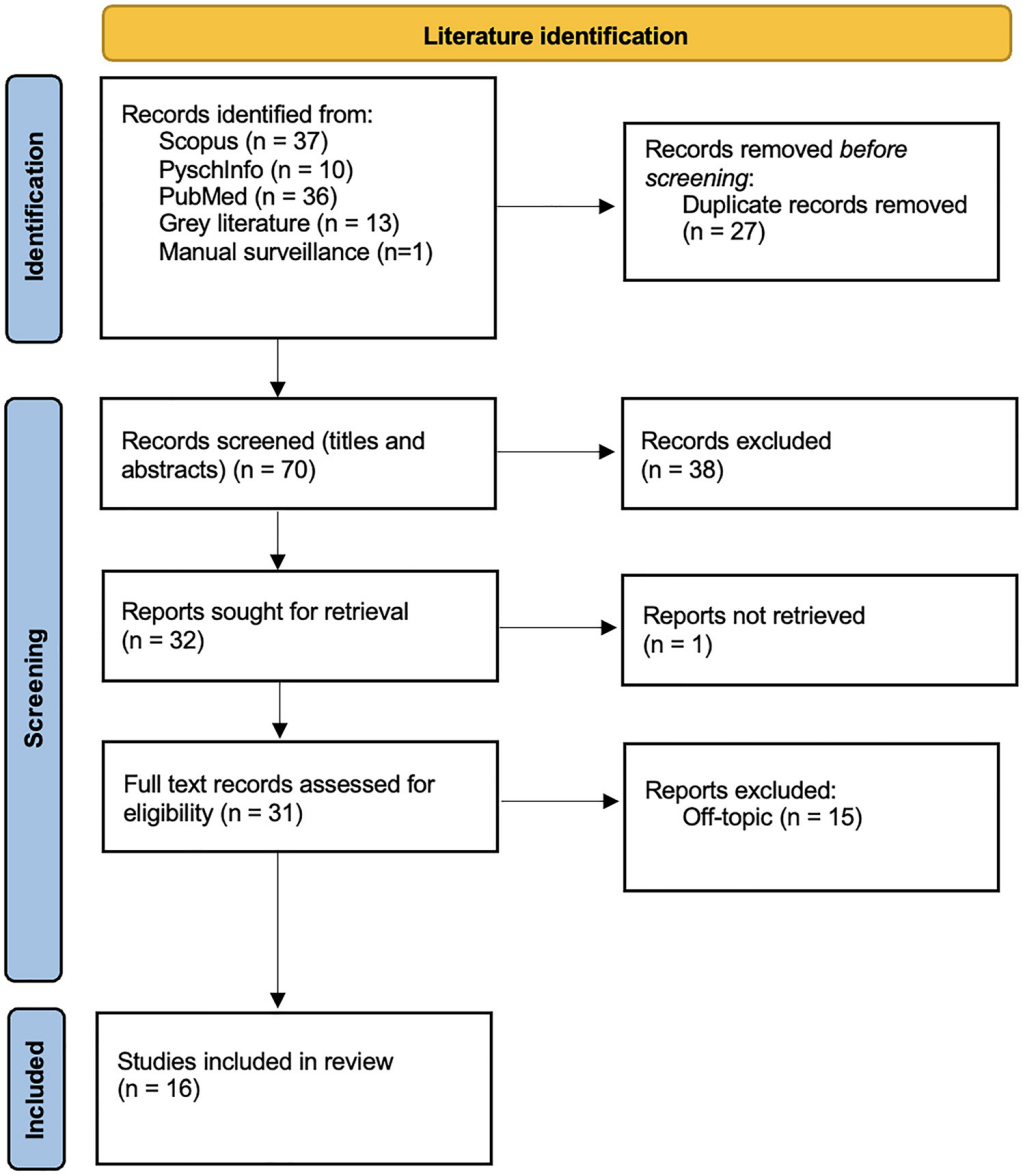
Search strategy and study selection PRISMA flow diagram

Inclusion criteria for eligible records were (1) published in English, (2) examined online gambling interfaces, (3) described or evaluated design features that could be interpreted as influencing user welfare in ways which reduce autonomy, transparency, or user welfare. We deliberately adopted a broad inclusion criteria, respecting the terminology as presented in each record rather than imposing our own operational definitions. This flexibility allowed us to capture a wide range of conceptually related design practices and was necessary due to the evolving and inconsistent nomenclature in this area. Following the literature search, the titles and abstracts of the resulting 70 records (27 duplicates removed from an initial 97) were screened by JM in line with the eligibility criteria, leading to 32 full texts sought for retrieval. One report was irretrievable, leading to a total of 31 full text records retrieved. Full text screening of these articles was performed by JM, JS, and JG, and involved regular meetings to discuss screening decisions until full agreement was met. In addition, one relevant article published after the original search period was identified through manual surveillance in May 2025 and met all inclusion criteria. Consequently, a final selection of 16 records were included in this review (Fig. 1).

## Results

A total of 16 articles published between 2018 and 2025 were included, comprising seven reports from grey literature institutions and nine articles published in peer-reviewed academic journals (see Table 1). Ten studies were conducted in the UK, with four in Australia, one in Belgium, and one jointly taking place in the UK and Australia (Table 1). Research methodologies varied between behavioural risk audits/mystery shopper exercises (Behavioural Insights Team (BIT), 2018, 2024; European Commission et al., 2022; Hing, Sproston, Brook, & Brading, 2018; Newall, Walasek, Ludvig, & Rockloff, 2022), qualitative surveys (Cemiloglu, Arden-Close, Hodge, & Ali, 2023; Citizens Advice, 2021, 2022; Hing et al., 2019), an online experiment (Cemiloglu, Gurgun, Arden-Close, Jiang, & Ali, 2023), and a field trial (BIT, 2021). Two papers focused on dark patterns in wider areas, mentioning their appearance in online gambling only in a singular annex (Citizens Advice, 2022), or briefly as part of a wider review (European Commission, 2022). These records were included in the present review as despite not focusing solely on online gambling, they nonetheless provided rare data concerning dark patterns in online gambling. A prior scoping study on the wider topic of nudges and gambling, which thematically included dark patterns (Fortier, Audette-Chapdelaine, Auger, & Brodeur, 2024) was not included in the present review as it did not specifically address dark patterns in depth and was limited to peer-reviewed research, thus not capturing the full scope of literature in this area. It should be noted that as almost all of the research to date has taken place in the UK and Australia, these results may not reflect experiences in other geographical areas.

**Table 1.** Characteristics table of included literature

Author(s)	Year	Region	Type	Objectives	Terminology	Methodology	Findings
Behavioural Insights Team	2018	UK	Grey literature report	To answer the research question: Can behavioural insights be used to reduce risky play in online environments?	“Nudges”	Review of existing operator practices, mystery shopper exercise, qualitative study (*n* = 18), two randomised control trials (*n* = 12,711), (*n* = 7,564)	Many features of remote gambling are likely to increase gambling involvement, online platforms are optimised to encourage greater amounts of money and time spent. There is need for research using real-world data and experimental designs
Behavioural Insights Team	2021	UK	Grey literature report	To answer the research question: Does making simple changes to how a deposit limit tool is presented to customers affects what kind of limits people set?	*No overarching term used*	Field trial (*n* = 45,000) of UK gambling customers	High anchors lead consumers to set higher deposit limits, removing these anchors may lead consumers to deposit less
Behavioural Insights Team	2022	UK	Grey literature report	To identify design features on operators' sites which may cause consumers to make choices not in their best interest	“Dark patterns”	Behavioural risk audit of 10 gambling and betting operator sites	Operator platforms contain several design features which lead customers astray from their intended behaviour
Behavioural Insights Team	2024	UK	Grey literature report	To identify relevant barriers and enablers to engagement with gambling management tools	“Dark patterns”	Behavioural risk audit of 10 gambling and betting operator sites	Gambling management tool design is inconsistent, hard to find, contains barriers, and focuses on users' individual responsibility rather than that of the operator
Cemiloglu, Arden-Close, et al.	2023	UK	Peer-reviewed journal article	To explore user perspectives of explainable persuasion in the context of online gambling	“Persuasive designs”	Survey (*n* = 250) of UK users of gambling platforms	Players were aware of the use, persuasive intent, and potential harm of various persuasive design techniques used in online gambling platforms, and agreed that explainable persuasion can still help users stay in control of their online experience, increase their positive attitude towards the online system, and keep them reminded of the potential side effects of persuasive interfaces
Cemiloglu, Gurgun, et al.	2023	UK	Peer-reviewed journal article	To explore the effectiveness of inoculation intervention as an approach to build resistance against persuasive interfaces on online gambling platforms	“Persuasive designs”	Online experiment (*n* = 240) of UK participants	Explainable persuasion increased awareness of the presence and risks of persuasive interfaces and strengthened user resistance to persuasive attempts
Citizens advice	2021	UK	Grey literature report	Uncover the ways in which ‘financial quicksand’ is built into the design of online gambling sites	“Financial quicksand”	Surveys (*n* = 2,015), (*n* = 2,110) of UK adults who had gambled online in the previous 3 months, mystery shopper exercise	Online gambling is easy to get into but difficult to get out of, design features of online gambling products is leading to harm, gambling controls are effective but underused, safer gambling requires safer defaults
Citizens advice	2022	UK	Grey literature report	To examine how specific changes to design can reduce serious harm in online gambling	“Tricks of the trade”	Survey (*n* = 2,014) of UK adults who had gambled online in the previous 3 months	Online gambling companies are failing to promote consumer best-interest decisions in several key aspects of platform design
European Commission	2022	Belgium	Grey literature report	To examine the prevalence of dark patterns across popular mobile apps and websites	“Dark patterns”	Mystery shopper exercise of 5 games/gambling platforms	Dark patterns were identified on games/gambling sites, most commonly in the form of ‘intermediate currencies’ and ‘nagging’
Gainsbury et al.	2020	Australia	Peer-reviewed journal article	To present a framework for how behavioural science principles can inform appropriate stakeholder actions to minimise internet gambling-related harms	“Persuasive designs”	N/A	Individual users, community groups, gambling industry, government and regulators, financial institutions, and researchers are all key stakeholders who can collaborate to reduce internet gambling-related harms
Hing et al.	2018	Australia	Peer-reviewed journal article	To document the range and structural features of financial inducements, and analyse their alignment with the harm minimisation and consumer protection goals of responsible gambling	*No overarching term used*	Scan of inducements offered on 30 race and sports betting brands, categorised into 15 generic types	Inducements were subject to numerous terms and conditions which were complex, difficult to find, and obscured by legalistic language. Play-through conditions of bonus bets were difficult to interpret and failed basic requirements for informed choice
Hing et al.	2019	Australia	Peer-reviewed journal article	To assess: whether the perceived attractiveness of inducements varies with the amount and type of information provided about their play-through conditions; bettors' comprehension of their true cost; and whether bettors' comprehension of their true cost varies with problem gambling severity	*No overarching term used*	Online survey (*n* = 299) of Australian sports bettors	Detailing key terms and conditions for an offer directly below the advertisement impacts negatively on its perceived attractiveness. 58% of bettors underestimated the additional amount they would need to bet to access any winnings from the bonus bet. No significant differences were found amongst gambler risk groups
Newall	2019	UK	Peer-reviewed journal article	“Gambling's ‘dark nudges’ are designed to exploit gamblers' biases. Gambling's dark nudges reveal the contradictions of industry-led responsible gambling initiatives, and show how stronger regulation is required to reverse gambling's spiralling public health costs”	“Dark nudges”	N/A	“While the gambling industry claims to support responsible gambling, the action of these same firms' dark nudges speak louder than words. Researchers, meanwhile, must do their best with what funding they have, and without access to gambling firms' proprietary data. The end result is an unreasonably large transfer of wealth from gamblers to the gambling industry”
Newall & Rockloff	2022	Australia	Peer-reviewed journal article	“Here, we argue that online gambling operators' actions are more consistent with sludge than nudge, and that sludge reduction shows more current promise for promoting safer gambling”	“Sludge”	N/A	“Although it would be beneficial to nudge gamblers toward safer choices, the prevention of both current and potential sludge practices should be of higher urgency in the agendas of those who want to promote safer gambling”
Newall et al.	2022	UK/Australia	Peer-reviewed journal article	To report on new research into whether gambling labels in the UK are more consistent with nudge or sludge	“Sludge”	Mystery shopper exercise	Gambling operators overwhelmingly use sludge instead of nudge strategies on gambling warning labels
Newall	2025	UK	Peer-reviewed journal article	To provide a taxonomy of online gambling platforms' deceptive design features	“Sludge, dark patterns, dark nudges, deceptive design”	N/A	“The complexity of on-line gambling platforms poses challenges for researchers, to understand what effects various design features have on behaviour, and also for policymakers, to ensure fairer outcomes for people. Increased awareness and collaboration are needed from many stakeholders to better understand deceptive design features' behavioral impacts and to give them the appropriate regulatory attention”

Following a narrative synthesis, the literature records were categorised across two overarching themes: *ambiguous terminology, and lack of available data obstructing research*. In addition, each example of a dark pattern identified on online gambling platforms to date was categorised in line with a transdisciplinary dark patterns framework (Table 2). Findings are presented below, with overarching themes described first, followed by the descriptive and tabulated categorisation of observed dark patterns in online gambling.

**Table 2.** Categorisation of dark patterns in online gambling identified within the literature records

Category	Type	Definition	Author(s)	Examples identified
Sneaking	Hiding information	*Hiding information or delaying the disclosure of information until later in the user journey that may have led them to making another choice*	BIT (2018, 2022, 2024), Newall et al. (2022), Hing et al. (2018)	Gambling management tools hidden behind small fonts and dark colours No net position or time spent displayed during gameplay Warning labels displayed in small fonts with lowest possible text boldness Terms and conditions of inducements are hard to find and difficult to interpret due to complex and dense language
Obstruction	Adding steps	*Creating additional points of unnecessary but required user interaction to perform a task*	BIT (2022, 2024)	Frictions and extra steps involved in setting gambling management tools
	Intermediate currencies	*Hide the true cost of a product by requiring the user to spend real money to purchase a virtual currency*	BIT (2022)	Coins or chips used instead of fiat currency during gameplay
	Creating barriers	*Preventing, abstracting or otherwise complicating a user task to disincentivise user action*	BIT (2022)	Minimum account balance required to withdraw funds
	Immortal accounts	*Make it difficult or impossible to delete a user account once it has been created*	BIT (2022), Citizens Advice (2021), European Commission (2022)	Difficult to find information on how to close an account Users need to contact customer support to close an account Accounts can be easily re-opened
Interface Interference	Visual prominence	*Places an element relevant to user goals in visual competition with a more distracting and prominent element*	BIT (2022, 2024)	Gambling management pages contain promotions, offers, and other gambling related cues
	Bad defaults	*Subverts the user's expectation that default settings will be in their best interest, instead requiring users to take active steps to change settings that may cause harm or unintentional disclosure of information*	BIT (2018, 2022, 2024), Citizens Advice (2021)	Deposit limit tools preset at high amounts or “no limit” by default Users automatically signed up to other brands/products upon account creation Default quick deposit and stake amounts above minimum required Default bet of previously staked amount Defaults for reality check set above minimum required
	Language inaccessibility	*Using unnecessarily complex language or a language not spoken by the user to decrease the likelihood the user will make an informed choice*	BIT (2022, 2024), Newall et al. (2022)	Gambling management pages are complicated and text heavy Warning labels are framed in a confusing and misleading manner
Forced Action	Nagging	*Distracting the user from a desired task to induce an action or make a decision the user does not want to make by repeatedly interrupting during normal interaction*	European Commission (2022)	Pop-up appears on each login inviting users to deposit
Social Engineering	Urgency	*Accelerating the user's decision-making process by demanding immediate or timely action*	BIT (2022)	After placing a bet, users are immediately prompted to bet again; presence of countdown clocks induce a sense of urgency
	Scarcity claims	*Subverts the user's expectation that information provided about a product's availability is accurate, instead pressuring the user to purchase a product without additional verification*	BIT (2022)	Time limited offers in marketing communications

### Ambiguous terminology

A key challenge across the literature is the inconsistent and ambiguous terminology with which observations of dark patterns in online gambling are described. Across the literature, dark patterns are referred to as “dark nudges” (Newall, 2019), “sludge” (Newall et al., 2022; Newall & Rockloff, 2022), “nudges” (BIT, 2018), “financial quicksand” (Citizens Advice, 2021), and “tricks of the trade” (Citizens Advice, 2022). Despite “dark patterns” being the dominant term in the wider deceptive design literature, only three papers in the present review use this term (BIT, 2022, 2024; European Commission et al., 2022). An additional three papers do not use any overarching term to describe the platform designs in question (BIT, 2021; Hing et al., 2018, 2019), while one vector of research in the literature focuses on mitigation strategies against “persuasive designs” (Cemiloglu, Arden-Close, et al., 2023; Cemiloglu, Gurgun, et al., 2023; Gainsbury et al., 2020). A more systematic approach to the taxonomy and identification of dark patterns can help to clarify the conceptual boundaries of the field and at which point persuasive designs venture into deceptive territory.

Newall (2025) presents an online gambling deceptive design taxonomy as a categorisation tool, within which they posit ‘dark nudges’ as being the core term which incorporates both sludge and dark patterns. Although dark nudges offers value as a concept closely associated with nudge theory, ‘dark patterns’ remains the most commonly used and widely recognised term in the broader literature. To maintain alignment with research in adjacent fields such as human-computer interaction and data privacy, it is important that the gambling field adopts terminology that facilitates interdisciplinary coherence.

### Lack of available data obstructing research

A recurring theme across the literature is researchers' frustrations with their lack of access to proprietary operator data, hindering their ability to conduct externally valid empirical research on the influence of dark patterns on consumer behaviour. BIT (2018, p. 107) a UK-based research consultancy that applies behavioural science to policy and regulatory contexts, note “although operators hold vast amounts of behavioural data on their customers, the amount of publicly available data is very limited…… it is likely that greater use and analysis of operator data is required to reveal important insights”. These concerns are echoed elsewhere in the literature, with Gainsbury et al. (2020, p. 5) remarking that “operators' commercial imperatives compete with their need for corporate social responsibility and duty of care”, and Newall (2019, p. 66), resigning that “researchers must do their best without access to gambling firms' proprietary data”.

In the sole field trial in the literature, BIT (2021) report that they were restricted by the gambling operator in how trial materials could be presented, interfering with the original design concept. The authors strongly recommended that “operators work with independent researchers to design, test, and share results from trials measuring the impact of safer gambling tools…. [and] that the Gambling Commission look to require operators to complete field trials aimed at independently evaluating their safer gambling practices” (p. 35).

### Categorisation of dark patterns in online gambling

The prevalence of dark patterns in online gambling is documented quantitatively across seven of the literature records. Using behavioural risk audits and mystery shopper exercises, dark patterns are found to be evident throughout the typical user experience on online gambling platforms. However, when specific examples of dark patterns are identified, they are often lacking in consistent definitions or classifications, preventing any connection to similar design practices identified on online gambling platforms, or in wider fields. Given the present inconsistencies and ambiguities in terminology, establishing a clear definition and classification system for dark patterns in online gambling is an essential next step towards providing a more structured foundation for future research.

To that end, all dark patterns identified in the included literature records are categorised below according to the Gray, Bielova, Santos, and Mildner (2024) dark patterns ontology (Table 2). We have chosen to adopt this particular framework to structure our findings as it offers a comprehensive classification system of dark patterns that synthesises and extends earlier work in the field. Additionally, compared to other available taxonomies (Brignull, Miquel, Rosenberg, & Offer, 2015; Mathur, Mayer, & Kshirsagar, 2021; Mills, Whittle, Ahmed, Walsh, & Wessel, 2023), the Gray et al. (2024) framework offers a balance between analytical depth and conceptual clarity, as well as a cohesive structure with which to support future empirical work across disciplinary boundaries, making it particularly well-suited to the needs of the present review. Instances of dark patterns reported in the included literature records were mapped to the categories of the ontology. Two researchers independently coded the articles, and discrepancies were resolved through discussion to ensure consistency. By adopting this classification method, the present review aims to structure the body of gambling research in alignment with the broader dark patterns literature, fostering a more cohesive body of knowledge. We envisage that this will provide clarity for future researchers who can use a similar structure to identify, define, and evaluate examples of dark patterns in online gambling, and for regulatory bodies who require a more systematic approach in defining key policy issues. As this is a relatively new and growing field of research, the examples categorised below are not expected to be a comprehensive documentation of all those in existence but will provide an outline and structure to those which have been identified to date.

#### Sneaking

Sneaking strategies *“hide, disguise or delay the disclosure of important information that, if made available to users, would cause a user to unintentionally take an action they would likely object to”* (Gray et al., 2024 p. 17). Sneaking strategies identified on online gambling platforms take the form of platform designs which hide information, particularly regarding safer gambling tools, inducements, warning labels and gameplay information. Gambling management pages are difficult to find, with links hidden behind small fonts and dark colours which could prevent users from finding such tools should they look for them (BIT, 2018, 2022, 2024). Given that only 16% of online gamblers report having used any gambling management tool (Gambling Commission, 2021a), platform designs which encourage, or at the very least do not discourage, the use of these tools can play a helpful role in increasing their uptake. Similarly, warning labels during gameplay are displayed in small fonts with lowest possible text boldness (Newall & Rockloff, 2022). Such designs are in sharp contrast with those of behaviours which benefit the platform designer, such as placing a bet, designed with large, colourful buttons in prominent positions on the webpage (BIT, 2022). Such disparity in design reflects the goals of the platform designer. By making areas of the user interface which benefit the operator (e.g., depositing, placing a bet) more salient than those which benefit the user (e.g., gambling management tools, information, terms and conditions) it is likely that users are influenced towards choices preferred by the operator. The lack of quantitative evidence on user attraction to different online gambling platform designs represents a distinct research gap in this area, which, when addressed, can provide important evidence to regulatory bodies to implement safer designs.

One literature record identifies a lack of information such as net position, current time of day, or time spent playing being displayed to users during gameplay (BIT, 2022). This design choice is in line with that of many physical casinos, who keep their venues well-lit at all hours and take care to not overtly indicate the time of the day to gamblers (Schüll, 2012). These strategies may facilitate “dark flow” states in which an individual gambles for long sessions due to a lack of perception of time during their gambling session (Lavoie & Main, 2019). During a recent consultation of members of the public and gambling operators, the Gambling Commission proposed net position and elapsed time to be displayed to consumers during gaming sessions (Gambling Commission, 2024b). Despite opposition from several operators, the proposal was implemented, with the Gambling Commission noting that it will provide consumers with greater transparency along with tools to monitor their spend and time. Initially mandated for slot games, the design change was later expanded to all casino games after an assessment found it led to a reduction in sessions lasting longer than an hour (Gambling Commission, 2024b).

Another area in which sneaking strategies have been identified is in financial inducements. Financial inducements incentivise gambling through offers such as free bets, deposit and sign-up bonuses, and, although not classified as dark patterns in and of themselves, Hing et al. (2018) identify several examples which venture into deceptive territory and mislead consumers. Inducements are often subject to terms and conditions which are difficult to find and interpret due to their inconspicuous placement and esoteric wording. Several offers contain conditions which may mask their true cost and expected value (e.g., bonus bets of $200 which require a play-through of $1,000), hidden within long pages of terms and conditions which are held on separate pages to the offers themselves. In a follow-up study, Hing et al. (2019) found that bettors often fail to comprehend the true cost of these inducements, and, when presented with clear information on their conditions, report a significant decrease in their perceived attractiveness. The presentation of information in clear formats and simple language can mitigate sneaking strategies by equipping consumers with the information required to make an informed, uninhibited decision on their gambling behaviours. Such design changes likely require regulatory mandates on the presentation of terms and conditions, as seen in Denmark, where they must be clearly presented by the operator alongside the initial inducement (Hing, Sproston, Brook, & Brading, 2015).

#### Obstruction

Obstruction strategies *“impede a user's task flow, making an interaction more difficult than it needs to be, dissuading a user from taking an action”* (Gray et al., 2024 p. 17). Obstruction strategies in online gambling are prevalent and come in several forms. Upon overcoming the initial challenges of locating gambling management tools, users are frequently met with additional hurdles. Setting deposit limits often involves navigating through several steps, making safer gambling behaviours less convenient to enact (BIT, 2024). Intermediate currencies such as coins or chips used instead of real money during gameplay can psychologically distance users from their actual spending (BIT, 2022). Another obstructive practice is the requirement of minimum account balances to withdraw funds, forcing users to either leave funds in their account or gamble the remaining balance rather than withdrawing it. This practice not only restricts user access to their own money, but also increases the likelihood of continued gambling (BIT, 2022).

“Immortal accounts” represent a particularly harmful obstruction tactic. Several papers within the literature report the difficulty with which intentions to close an account can be carried out. Users are often required to contact customer support in order to permanently close their account, with no clear information on how to do so, while even after an account has been closed they can be reactivated with minimal effort, undermining the decision of users to stop gambling (BIT, 2022; Citizens Advice, 2021; European Commission et al., 2022). This practice can trap users in a cycle where they cannot fully disengage from the platform, and aligns with similar practices observed in social media account settings (Bösch et al., 2016). Users also face frictions when attempting to unsubscribe from marketing communications, with unclear options and multi-step processes hindering the efforts of users wishing to limit their exposure or disengage entirely from promotional messages (BIT, 2022).

#### Interface interference

Interface interference strategies *privilege specific actions over others through manipulation of the user interface, thereby confusing the user or limiting discoverability of relevant action possibilities* (Gray et al., 2024 p. 17)*.* Interface interference strategies are the most documented form of dark patterns in online gambling, with “bad defaults” being the most prevalent. Bad defaults exploit the cognitive biases of inertia and anchoring, whereby humans are strongly influenced by initial reference points, and tend to stick with pre-selected options (Madrian & Shea, 2001). Online gambling platforms exhibit several examples of defaults and anchors against the best interest of the consumer. For example, deposit limit tools often feature dropdown menus with high amounts such as £5,000 as the first option, and free-text boxes that allow deposit limits up to £10 million, while both deposit and stake boxes are prepopulated with amounts that exceed the minimum required (BIT, 2018, 2024; Citizens Advice, 2021). Other examples include default reality checks set at extended intervals, such as four hours when 15 min is the minimum, and automatic enrolment to other gambling brands or products upon account creation (BIT, 2022). Given the evidence of the strong influence of defaults on decision making, these practices are likely to influence users towards riskier behaviours. A field trial conducted by BIT (2021) found that redesigning deposit limit tools to remove high default options and replace them with a free-text box reduced deposit limits by 45%. Despite these encouraging results, this simple, high-impact design change has yet to be implemented by any online gambling platform to date.

Other interface interference tactics include language inaccessibility and visual prominence. Warning labels are often framed in confusing or misleading ways, potentially reducing their effectiveness in communicating risks (Newall et al., 2022). Safer gambling pages are typically designed with dense, text-heavy layouts and lack visual appeal, making it more difficult for users to fully comprehend the steps required to limit their gambling activity (BIT, 2022). Paradoxically, gambling management pages often contain promotions, offers, and other gambling-related cues which appear to undermine the intended purpose of such pages (BIT, 2022, 2024). On landing pages, links to safer gambling tools are relegated to the bottom, while offers, advertisements, and gambling prompts dominate the top of the page. Bet slips similarly emphasize placing bets as the most appealing option, using large, colourful buttons to draw attention (BIT, 2022).

#### Forced action

Forced action strategies *require users to knowingly or unknowingly perform an additional and/or tangential action or information to access specific functionality, preventing them from continuing their interaction with a system without performing that action* (Gray et al., 2024, p. 18). To date, only one instance of a forced action dark pattern has been documented within the gambling literature. In a wide-ranging investigation into dark patterns on online platforms, the European Commission (2022) document “nagging” behaviours on gambling sites in the form of pop-ups which appear on each login which invite users to deposit funds to their accounts. Although evidence of forced action strategies is limited, future research which systematically documents dark patterns on online gambling platforms may reveal further examples.

#### Social engineering

Social engineering strategies *present options or information that causes a user to be more likely to perform a specific action based on their individual and/or social cognitive biases, thereby leveraging a user's desire to follow expected or imposed social norms* (Gray et al., 2024 p. 19). Social engineering strategies are documented within one of the literature records. BIT (2022) describe prompts to bet again immediately after placing a bet, with the presence of countdown clocks potentially inducing a sense of urgency. The authors also report time limited offers in marketing communications using urgency claims to influence behaviour. Similar examples have been identified on online shopping websites (Mathur et al., 2019), where many countdown timers were found to be fraudulent, simply resetting after timeout with the “limited time” offer remaining valid. No research to date has tested the validity of such messages on online gambling sites, although due to the nature of many such offers which typically countdown to live sporting events, it is unlikely that such deception will be seen on a similar scale. Urgency tactics have also been documented in television gambling advertising during the football World Cup, including the use of “flash odds” which are marketed as enhanced prices available for a limited time, typically marketed during the half time break of the match to which the odds relate (Newall, Thobhani, Walasek, & Meyer, 2019).

## Discussion

The present scoping review is the first to systematically examine the literature on dark patterns in online gambling. Dark patterns have been documented across key user interactions in online gambling such as deposit and withdrawal processes (BIT, 2018, 2022), gambling management tools (BIT, 2018, 2022, 2024), and account closure (BIT, 2022; Citizens Advice, 2021; European Commission et al., 2022). However, with the exception of a singular field trial (BIT, 2021), there is not yet any evidence on how dark patterns influence consumer behaviour, underlining the critical need for behavioural research in this field.

Dark patterns research can be conceptualised into three broad areas: taxonomy, identification, and evaluation. In the field of online gambling, research has focussed on the identification of dark patterns through prevalence studies, while the application of taxonomies or frameworks have, until the present review, been largely overlooked. By categorising dark patterns in online gambling within the Gray et al. (2024) framework, our synthesis offers a structure with which future research can identify, document, and evaluate dark patterns in a more comprehensive and universal manner.

Future research should prioritise the development of experimental studies to empirically quantify the influence of dark patterns on behaviour. Given the unlikely cooperation of gambling operators, such studies much rely on independent lab experiments which as closely as possible mirror real-life gambling platforms. These experiments can provide robust, causal evidence of the influence of dark patterns on user behaviour, as well as offer the opportunity to test the effectiveness of design interventions aimed at harm reduction. Exploring individual differences in susceptibility to dark patterns is also an important next step. Variables such as problem gambling severity measures, age, digital literacy, and sociodemographic characteristics should be analysed to determine predictors of susceptibility, enabling the design of targeted harm-reduction interventions to better protect at-risk users from gambling harms. The current dearth of data on the influence or impact of dark patterns on consumer behaviour reflects a core challenge to research in this domain, in that obtaining externally valid behavioural data requires access to proprietary operator data, or else the creation of near-naturalistic lab experiments (Newall, 2025). Access to such data is typically restricted due to the inherent conflict between the financial incentives of gambling industry operators and the implementation of safer gambling practices. A clear regulatory mandate for data-sharing between operators and researchers would open the doors to robust, empirical insights across key demographics, though such openness will likely only result from regulatory action, as the findings of this research may lead to safer, and therefore less profitable, platform designs. Until such mandates are introduced, researchers must be innovative in their experimental designs to uncover important behavioural insights.

There is also a need for more systematic prevalence studies of dark patterns in online gambling. Existing studies in other domains, such as that of Mathur et al. (2021) in online shopping, provide high-quality evidence on the pervasiveness of dark patterns at scale. Similar studies in online gambling would offer a comprehensive picture of the prevalence of dark patterns, enabling researchers and regulators to better prioritise research and intervention efforts. Future research should also examine consumer perceptions of dark patterns, focusing on how users identify, interpret, and react to these designs. An insight into user perspectives can guide the development of consumer empowerment tools, such as educational campaigns or interface design modifications that promote transparency and autonomy.

The close interrelation of behavioural science and dark patterns offers a framework with which potential countermeasures can be explored to foster a more user-friendly online gambling environment. The integration of counter-nudges or “light patterns” (Luguri & Strahilevitz, 2021) can provide such an environment, protecting users against the interactions between their own cognitive biases and dark patterns which are specifically designed to target and exploit such biases. Subtle design changes such as making gambling management tools more prominent and easier to find, timely reminders designed to interrupt “dark flow” gambling sessions, or defaults which benefit the consumer rather than the operator, can play a role in creating a safer online gambling environment. The removal of sludge in areas such as withdrawal and account closure processes can also aid consumers in providing them with the tools required to perform their intended actions unimpeded.

Dark patterns do not necessarily stem from designers with ill intent, and are likely the byproduct of data-driven optimisation processes such as a/b testing, in which different designs are tested at mass scales where the most profitable (and therefore most loss-heavy for consumers) design is retained (Narayanan, Mathur, Chetty, & Kshirsagar, 2020; Newall, 2019). Moreover, not all dark patterns are universally experienced as harmful (Mills, 2024) argues that this necessitates regulatory flexibility, advocating for a principles-based approach to the regulation of choice architecture, supported by behavioural auditing. In the domain of online gambling, such an approach holds promise, though the realities of identifying and policing breaches of such principles remains uncertain. Whether gambling firms intentionally manipulate and mislead their consumers remains uncertain, but what is clear is that dark patterns are perceived as unethical by the public (Maier, 2019), and, when presented with evidence of unsafe designs, regulators have demonstrated a willingness to intervene. In recent years, platform designs such as auto-play on slots, losses disguised as wins, reverse withdrawals and fast slot speeds have been banned (Gambling Commission, 2021b), after evidence found that they led to unnecessary gambling harms. The key issue is that under current legislation, gambling regulators are forced to be reactive rather than proactive, resulting in prolonged exposure to harm for vulnerable consumers. Existing regulatory models in other sectors may offer a blueprint for more anticipatory governance. For example, the UK Financial Conduct Authority's (FCA) *Consumer Duty* (FCA, 2022) requires firms to demonstrate that they are acting in the best interests of consumers. This includes proactively identifying and mitigating foreseeable harm, and ensuring that consumers receive communications they can understand and services that meet their needs, principles that could be meaningfully adapted to online gambling regulation. Such an approach could lessen the reliance on difficult-to-conduct academic research, by shifting the burden of proof onto operators to demonstrate that their platforms are designed to support consumer wellbeing, thereby fostering a more proactive and preventative regulatory environment.

As gambling activities continue to shift from physical bookmakers and towards online platforms, consumers are exposed to greater risks of harm due to personalised profiling, real-time behavioural tracking, and the use of sophisticated, behaviourally informed platform designs. Online gambling is a strong predictor of harm (Allami et al., 2021), and these digital environments can be personally tailored to exploit cognitive biases, nudging vulnerable users toward prolonged and riskier gambling activities. Gambling operators who host such addictive technologies have a responsibility to protect consumers, despite the inherent conflict between their revenue model and the implementation of such protections. With access to vast amounts of user data, gambling operators are well positioned to design environments that support informed consumer choice. Embedding this responsibility into formal responsible gambling mandates, supported by proactive regulatory action, is an essential next step to ensure that consumer protection becomes a core principle in a continually growing industry with significant potential for harm.

## Conclusions

Dark patterns are deeply embedded into the user experience of online gambling platforms, and may influence consumer behaviour in ways that may lead to increased losses and prolonged engagement. However, inconsistencies in how these deceptive design practices are defined and identified, coupled with a lack of empirical evidence on their direct impact, have contributed to a fragmented literature and slow regulatory response. As online gambling platforms continue to evolve and grow in sophistication, there is an urgent need for robust research which quantifies the influence of dark patterns on consumer behaviour. Such research can inform evidence-based regulatory action to safeguard vulnerable consumers from gambling harms. Strong regulatory action should require operators to embed consumer welfare into the core of platform design to prevent the exploitation of cognitive vulnerabilities in an increasingly data-driven digital environment.

## Appendix

**Table A1.** Grey literature institutions included in search (Behavioural Insights Team and Citizens Advice, 2024)

1. Advertising Standards Authority (ASA)
2. Australian Competition and Consumer Commission
3. Behavioural Insight Team
4. Canada Competition Bureau
5. Canadian Office of Consumer Affairs
6. Citizens Advice
7. Competition and Markets Authority
8. Consumer Reports Advocacy
9. Danish Competition and Consumer Authority
10. Department for Business and Trade
11. Department for Science, Innovation & Technology
12. European Commission
13. European Consumer Centre Sweden
14. Financial Conduct Authority (FCA)
15. Federal Trade Commission
16. Norwegian Consumer Council
17. Gambling Commission
18. Government Equalities Office and Equality and Human Rights Commission
19. International Consumer Protection and Enforcement Network (ICPEN)
20. Konsumentverket (Swedish Consumer Agency)
21. Money and Mental Health Policy Institute
22. Netherlands Authority for Consumers and Markets
23. New America Foundation
24. Organisation for Economic Co-operation and Development (OECD)
25. Ofcom
26. Parentzone
27. The Committee of Advertising Practice
28. The Money Advice Service
29. Which?
